# Development and Implementation of the AIDA International Registry for Patients With VEXAS Syndrome

**DOI:** 10.3389/fmed.2022.926500

**Published:** 2022-07-11

**Authors:** Antonio Vitale, Valeria Caggiano, Francesca Della Casa, José Hernández-Rodríguez, Micol Frassi, Sara Monti, Abdurrahman Tufan, Salvatore Telesca, Edoardo Conticini, Gaafar Ragab, Giuseppe Lopalco, Ibrahim Almaghlouth, Rosa Maria R. Pereira, Derya Yildirim, Marco Cattalini, Achille Marino, Teresa Giani, Francesco La Torre, Piero Ruscitti, Emma Aragona, Ewa Wiesik-Szewczyk, Emanuela Del Giudice, Petros P. Sfikakis, Marcello Govoni, Giacomo Emmi, Maria Cristina Maggio, Roberto Giacomelli, Francesco Ciccia, Giovanni Conti, Djouher Ait-Idir, Claudia Lomater, Vito Sabato, Matteo Piga, Ali Sahin, Daniela Opris-Belinski, Ruxandra Ionescu, Elena Bartoloni, Franco Franceschini, Paola Parronchi, Amato de Paulis, Gerard Espinosa, Armin Maier, Gian Domenico Sebastiani, Antonella Insalaco, Farhad Shahram, Paolo Sfriso, Francesca Minoia, Maria Alessio, Joanna Makowska, Gülen Hatemi, Nurullah Akkoç, Francesca Li Gobbi, Antonio Gidaro, Alma Nunzia Olivieri, Sulaiman M. Al-Mayouf, Sükran Erten, Stefano Gentileschi, Ibrahim Vasi, Maria Tarsia, Ayman Abdel-Monem Ahmed Mahmoud, Bruno Frediani, Musa Fares Alzahrani, Ahmed Hatem Laymouna, Francesca Ricci, Fabio Cardinale, Karina Jahnz-Rózyk, Gian Marco Tosi, Francesca Crisafulli, Alberto Balistreri, Marília A. Dagostin, Mahmoud Ghanema, Carla Gaggiano, Jurgen Sota, Ilenia Di Cola, Claudia Fabiani, Henrique A. Mayrink Giardini, Alessandra Renieri, Alessandra Fabbiani, Anna Carrer, Monica Bocchia, Federico Caroni, Donato Rigante, Luca Cantarini

**Affiliations:** ^1^Department of Medical Sciences, Surgery and Neurosciences, Research Center of Systemic Autoinflammatory Diseases and Behçet's Disease Clinic, University of Siena, Siena, Italy; ^2^Section of Clinical Immunology, Department of Translational Medical Sciences, University of Naples Federico II, Naples, Italy; ^3^Vasculitis Research Unit and Autoinflammatory Diseases Clinical Unit, Department of Autoimmune Diseases, Hospital Clinic of Barcelona, Institut d'Investigacions Biomèdiques August Pi i Sunyer, University of Barcelona, Barcelona, Spain; ^4^Rheumatology and Clinical Immunology Unit, Department of Clinical and Experimental Sciences, ASST Spedali Civili, University of Brescia, Brescia, Italy; ^5^Rheumatology Department, IRCCS Policlinico S. Matteo Foundation, Pavia, Italy; ^6^Department of Experimental Medicine, University of Pavia, Pavia, Italy; ^7^Department of Internal Medicine and Rheumatology, Gazi University, Ankara, Turkey; ^8^Rheumatology and Clinical Immunology Unit, Internal Medicine Department, Faculty of Medicine, Cairo University, Giza, Egypt; ^9^Faculty of Medicine, Newgiza University (NGU), Giza, Egypt; ^10^Rheumatology Unit, Department of Emergency and Organ Transplantation, University of Bari, Bari, Italy; ^11^Rheumatology Unit, Department of Medicine, College of Medicine, King Saud University, Riyadh, Saudi Arabia; ^12^College of Medicine Research Center, College of Medicine, King Saud University, Riyadh, Saudi Arabia; ^13^Rheumatology Division, Faculdade de Medicina, Hospital das Clínicas HCFMUSP, Universidade de São Paulo, São Paulo, Brazil; ^14^Pediatric Clinic, Spedali Civili di Brescia, University of Brescia, Brescia, Italy; ^15^Unit of Pediatric Rheumatology, ASST Gaetano Pini-CTO, Milan, Italy; ^16^Department of Clinical Sciences and Community Health, Research Center for Adult and Pediatric Rheumatic Diseases, ASST G. Pini-CTO, University of Milan, Milan, Italy; ^17^Department of Pediatrics, Pediatric Rheumatology Center, Ospedale “Giovanni XXIII”, Azienda Ospedaliera-Universitaria Consorziale Policlinico, Bari, Italy; ^18^Rheumatology Unit, Department of Biotechnological and Applied Clinical Sciences, University of L'Aquila, L'Aquila, Italy; ^19^Division of Gastroenterology, Ospedali Riuniti Villa Sofia-Vincenzo Cervello, Palermo, Italy; ^20^Department of Internal Medicine, Pulmonology, Allergy and Clinical Immunology, Central Clinical Hospital of the Ministry of National Defence, Military Institute of Medicine, Warsaw, Poland; ^21^Department of Maternal Infantile and Urological Sciences, Polo Pontino, Sapienza University of Rome, Rome, Italy; ^22^Joint Academic Rheumatology Program, 1st Department of Propedeutic Internal Medicine, School of Medicine, National and Kapodistrian University of Athens, Athens, Greece; ^23^Rheumatology Unit, Department of Medical Sciences, Azienda Ospedaliero-Universitaria S. Anna–Ferrara, University of Ferrara, Ferrara, Italy; ^24^Department of Experimental and Clinical Medicine, University of Florence, Florence, Italy; ^25^University Department Pro.Sa.M.I. “G. D'Alessandro”, University of Palermo, Palermo, Italy; ^26^Rheumatology, Immunology and Clinical Medicine Unit, Department of Medicine, Università Campus Bio-Medico di Roma, Selcetta, Italy; ^27^Department of Precision Medicine, Università Degli Studi Della Campania Luigi Vanvitelli, Naples, Italy; ^28^Pediatric Nephrology and Rheumatology Unit, AOU G Martino, Messina, Italy; ^29^Research Laboratory, Biodiversity, Biotechnology, Environment and Sustainable Development, Faculty of Sciences, M'Hamed Bougara University, Boumerdes, Algeria; ^30^Unità Operativa (UO) Reumatologia, AO Ordine Mauriziano, Turin, Italy; ^31^Department of Immunology, Allergology, and Rheumatology, Antwerp University Hospital, University of Antwerp, Antwerp, Belgium; ^32^Rheumatology Unit, Department of Medical Sciences, University and Azienda Ospedaliera-Universitaria of Cagliari, Cagliari, Italy; ^33^Division of Rheumatology, Department of Internal Medicine, Sivas Cumhuriyet University, Sivas, Turkey; ^34^Department of Internal Medicine and Rheumatology, Carol Davila University of Medicine and Pharmacy, Bucharest, Romania; ^35^Rheumatology Unit, Department of Medicine, University of Perugia, Perugia, Italy; ^36^Center for Basic and Clinical Immunology Research (CISI), WAO Center of Excellence, University of Naples Federico II, Naples, Italy; ^37^Rheumatology Unit, Department of Medicine, Central Hospital of Bolzano, Bolzano, Italy; ^38^Unità Operativa Complessa (U.O.C.) Reumatologia, Ospedale San Camillo-Forlanini, Rome, Italy; ^39^Division of Rheumatology, Ospedale Pediatrico Bambino Gesù, IRCCS (European Reference Network for Immunodeficiency, Autoinflammatory and Autoimmune Diseases Center), Rome, Italy; ^40^Behcet's Disease Unit, Rheumatology Research Center, Shariati Hospital, Tehran University of Medical Sciences, Tehran, Iran; ^41^Rheumatology Unit, Department of Medicine DIMED, University of Padova, Padova, Italy; ^42^Pediatric Rheumatology, Fondazione IRCCS (Istituto di ricovero e cura a carattere scientifico) Ca' Granda Ospedale Maggiore Policlinico, Milan, Italy; ^43^Pediatric Rheumatology Unit, Department of Translational Medical Sciences, University of Naples Federico II, Naples, Italy; ^44^Department of Rheumatology, Medical University of Lodz, Lodz, Poland; ^45^Division of Rheumatology, Department of Internal Medicine, Behçet's Disease Research Center, Istanbul University–Cerrahpasa, Istanbul, Turkey; ^46^Division of Rheumatology, Department of Internal Medicine, School of Medicine, Manisa Celal Bayar University, Manisa, Turkey; ^47^Rheumatology Unit, San Giovanni di Dio Hospital, Firenze, Italy; ^48^Department of Biomedical and Clinical Sciences 'Luigi Sacco', University of Milan, Milan, Italy; ^49^Department of Woman, Child and of General and Specialized Surgery, University of Campania “Luigi Vanvitelli”, Naples, Italy; ^50^King Faisal Specialist Hospital and Research Center, Riyadh, Saudi Arabia; ^51^Department of Rheumatology, Ankara City Hospital, Ankara, Turkey; ^52^Unit of Rheumatology, Azienda Ospedaliero-Universitaria Senese, Siena, Italy; ^53^Department of Medicine, College of Medicine, King Saud University, Riyadh, Saudi Arabia; ^54^Ophthalmology Unit, Department of Medicine, Surgery and Neurosciences, University of Siena, Siena, Italy; ^55^Bioengineering and Biomedical Data Science Lab, Department of Medical Biotechnologies, University of Siena, Siena, Italy; ^56^Medical Genetics, Department of Medical Biotechnologies, University of Siena, Siena, Italy; ^57^Department of Medical Biotechnologies, Med Biotech Hub and Competence Center, University of Siena, Siena, Italy; ^58^Genetica Medica, Azienda Ospedaliero-Universitaria Senese, Siena, Italy; ^59^Unit of Hematology, Azienda Ospedaliera Universitaria Senese, University of Siena, Siena, Italy; ^60^Department of Life Sciences and Global Health, Fondazione Policlinico Universitario A. Gemelli IRCCS, Rome, Italy; ^61^Rare Diseases and Periodic Fevers Research Centre, Università Cattolica del Sacro Cuore, Rome, Italy

**Keywords:** autoinflammatory diseases, clinical management, precision medicine, rare diseases, research, treatment

## Abstract

**Objective:**

The aim of this paper is to present the AutoInflammatory Disease Alliance (AIDA) international Registry dedicated to Vacuoles, E1 enzyme, X-linked, Autoinflammatory, Somatic (VEXAS) syndrome, describing its design, construction, and modalities of dissemination.

**Methods:**

This Registry is a clinical, physician-driven, population- and electronic-based instrument designed for the retrospective and prospective collection of real-life data. Data gathering is based on the Research Electronic Data Capture (REDCap) tool and is intended to obtain real-world evidence for daily patients' management. The Registry may potentially communicate with other on-line tools dedicated to VEXAS syndrome, thus enhancing international collaboration and data sharing for research purposes. The Registry is practical enough to be easily modified to meet future needs regarding VEXAS syndrome.

**Results:**

To date (April 22^nd^, 2022), 113 Centers from 23 Countries in 4 continents have been involved; 324 users (114 Principal Investigators, 205 Site Investigators, 2 Lead Investigators, and 3 data managers) are currently able to access the registry for data entry (or data sharing) and collection. The Registry includes 4,952 fields organized into 18 instruments designed to fully describe patient's details about demographics, clinical manifestations, symptoms, histologic details about skin and bone marrow biopsies and aspirate, laboratory features, complications, comorbidities, therapies, and healthcare access.

**Conclusion:**

This international Registry for patients with VEXAS syndrome will allow the achievement of a comprehensive knowledge about this new disease, with the final goal to obtain real-world evidence for daily clinical practice, especially in relation to the comprehension of this disease about the natural history and the possible therapeutic approaches. This Project can be found on https://clinicaltrials.gov NCT05200715.

## Introduction

VEXAS (Vacuoles, E1 enzyme, X-linked, Autoinflammatory, Somatic) syndrome is a recently recognized pathological entity first reported in December 2020. VEXAS represents a monogenic autoinflammatory condition caused by acquired somatic mutations in UBA1, gene encoding one of the two E1 enzyme isoforms that initiates ubiquitylation in cell's cytoplasm. Unlike other genetic autoinflammatory syndromes, which are due to germline mutations in most of cases, VEXAS syndrome is caused by acquired variants in blood cells precursors, especially myeloid progenitors (1). Because of its recent identification, VEXAS clinical features, complications, outcome, and treatment strategies are not fully established at current. However, it is clearly characterized by prominent inflammation involving almost all organs and tissues with highly increased inflammatory markers. The skin, eyes, lungs, joints, and gastrointestinal system are frequently affected by the disease, with a quite protean range of inflammatory manifestations. Besides these clinical features, which account for a common ground with other “historical” autoinflammatory diseases, hematologic involvement represent the most typical disease manifestation. Indeed, hematological involvement is observed in at least 50% of patients, with myelodysplastic syndrome (MDS) representing the most frequent bone marrow affection. Monoclonal gammopathy with unknown significance (MGUS), macrocytic anemia with normal vitamin B12 and folate levels, leukopenia, and thrombocytopenia are additional hematological features. Noteworthy, marked cytoplasmic vacuolization in hematopoietic precursors are often observed in bone marrow aspirate smears, which represent a good diagnostic clue. Vacuoles are generally identified in erythroid and myeloid precursors (blasts, promyelocytes, and pronormoblasts), but are also observed in eosinophils, monocytes, plasma cells, and megakaryocytes to a lesser degree (2).

At current there is no data about the effective epidemiological burden of VEXAS syndrome, which as to be consider a rare disease based on the current prevalence. Therefore, as with other rare diseases, VEXAS syndrome may benefit from patient registries capable of leading to a better understanding of the disease in a relatively short time. In particular, patient registries are overcoming current research approaches, especially for rare diseases. International registries have the potential to recruit a wide number of patients worldwide and follow enrolled subjects for very long periods of time. The importance of patient registries in the field of rare diseases is shown by the relevance provided by the European Union (EU) to this online tool, making available specific guidelines for high-quality registries (3–5).

Regarding autoinflammatory diseases, the AutoInflammatory Disease Alliance (AIDA) has already developed and launched eight international registries for patients with many rare autoinflammatory diseases (6–8). The AIDA Project has already allowed the construction of an international Network of physicians and researchers interested in putting together information to expand current evidence about monogenic autoinflammatory diseases, Still's disease, Schnitzler's syndrome, Behçet's disease, periodic fever, aphthous stomatitis, pharyngitis, cervical adenitis (PFAPA) syndrome, non-infectious uveitis, non-infectious scleritis, and undifferentiated systemic autoinflammatory diseases (USAIDs). For more details, the AIDA Network may be accessed at the following website: https://aidanetwork.org/en/.

Based on the experience of the AIDA Network in developing registries for rare autoinflammatory diseases, an international patient Registry specifically dedicated to VEXAS syndrome has been developed. This manuscript aims to illustrate the objectives, design, methodology and modalities of diffusion underlying the development and activation of the VEXAS Registry.

## Materials and Methods

### Study Design

The AIDA Registry presented in this work has been created as an international, clinical, physician-driven, population- and electronic-based registry dedicated to patients diagnosed with VEXAS syndrome.

Data collection includes a retrospective phase, for data gathered up to the time of the enrolment in the Registry, and a prospective phase for progressive data available starting from the time of the enrolment. The prospective phase requires the collection of at least one follow-up visit per year. However, prospective data collection should be performed whenever a change in the treatment strategy occurs.

The Registry is designed to collect demographic, genetic, clinical, laboratory and treatment data starting since the disease onset. These data will be essentially derived from the routine follow-up visits performed to guarantee the best standard of care, while no additional information will be required. In the same way, none of the treatment choices and drug adjustments will be influenced by the participation to this Project. Indeed, physicians' clinical judgement based on current evidence represents the only factor capable of determining the therapeutic management of the patient.

The access is open for all Centers dealing with the management, diagnosis, and treatment of VEXAS syndrome. The Centers that would like to participate, may join the AIDA Network by contacting the Promoter or sending an email to the AIDA Team by writing to support@aidaregistry.org or using a specific form, that may be found at the following page: https://aidanetwork.org/en/aida.

All clinical specialities are included in the AIDA Network; the location and the type of practice setting do not influence the inclusion in this Project. As data inserted in the Registry are usually included in the standard management of VEXAS patients, no costs or financial fees are settled. As an essential prerequisite for the inclusion in this Project, each Center must obtain the approval from the local ethics committee. Also, it is essential to identify a Principal Investigator for the local coordination of the study and at least a Site Investigator, who will manage documentation and take care of data collection. Both the Principal Investigator and the Site Investigator will receive the credentials to enter the Registry and start patients' enrolment after having expressed the formal intention to participate in the VEXAS Registry.

### Registry Objectives

The Registry for patients with VEXAS syndrome is primarily aimed at gathering information from the larger number of patients as possible. A large cohort of patients is critical to obtain solid evidence from data analysis and transfer the results into daily clinical practice. A further objective of the Registry is to learn about VEXAS syndrome in detail and in a rapid manner, avoiding the delays that would inevitably result from traditional clinical research, which generally relies on limited study populations available at a single research center.

Additional objectives of this Project are: (I) to fully characterize the wide spectrum of inflammatory manifestations and their frequency; (II) to eventually identify different disease subtypes; (III) to describe mutations that will be found in the *UBA1* gene, to differentiate among pathogenic or likely pathogenic variants from benign polymorphisms; (IV) to search for genotype-phenotype associations; (V) to study the influence of other mutations on genes associated with monogenic autoinflammatory diseases; (VI) to identify any pathognomonic features to facilitate diagnosis; (VII) to develop classification criteria and diagnostic algorithms to be applied in clinical practice to select patients for genetic assessment; (VIII) to identify variables capable of distinguishing VEXAS patients from other mimicking diseases; (IX) to fully understand the possible spectrum of haematologic disorders; (X) to describe hematologic and non-hematologic complications occurred in the long-term; (XI) to better characterize information from bone marrow biopsy and aspirate; (XII) to search for prognostic factors able to select patients with a higher probability to develop complications; (XIII) to recognize predisposing factors and triggers associated with the onset and disease's exacerbations, quantifying and stratifying the severity of the features; (XIV) to describe treatment attempts, taking in to account their efficacy as a whole and the impacts on the different aspect of the disease; (XV) to report the safety profile of single treatment approaches in VEXAS patients; (XVI) identifying the better treatment approach tapered on the patient's features and disease characteristics; (XVII) to carefully study treatment dosages and their changes to develop standardized treatment protocols; (XVIII) to asses any influence of the environmental background and the ethnic origin on the VEXAS syndrome phenotype; (XIX) to assess any impact of the socioeconomic status in terms of access to healthcare and patients' absenteeism due to the disease; (XX) to monitor the cardiovascular risk in such patients; (XXI) to monitor the causes of death in VEXAS syndrome.

Other pioneering studies will be eventually designed according to the unmet needs arising from patients' management over time.

Table 1 summarizes primary and additional objectives of this Registry.

**Table 1:** Objectives considered for the implementation of the AIDA registry for patients with VEXAS (vacuoles, E1 enzyme, X-linked, autoinflammatory, somatic) syndrome.

Primary objectives	To collect as much real-world data from a large cohort of patients enrolled with an international basis
	To avoid the time delay associated with the traditional clinical research in obtaining a comprehensive knowledge and awareness about VEXAS syndrome
Additional objectives	To fully characterize the wide spectrum of inflammatory manifestations and their frequency
	To eventually identify different disease subtypes
	To differentiate among pathogenic and likely pathogenic variants from benign polymorphisms that will be found in the *UBA1* gene
	To search for any genotype-phenotype associations
	To study the influence of other mutations on genes associated with monogenic autoinflammatory diseases
	To identify any pathognomonic features able to facilitate diagnosis
	To develop classification criteria and diagnostic algorithms to be applied in clinical practice to select patients for genetic assessment
	To identify variables capable of distinguishing VEXAS patients from other mimicker diseases
	To fully understand the possible spectrum of hematological disorders associated with VEXAS syndrome
	To describe hematologic and non-hematologic complications occurring in the long-term
	To better characterize information from bone marrow biopsy and aspirate
	To search for prognostic factors able to select patients with a higher probability to develop complications
	To recognize predisposing factors and triggers associated with the onset and disease's exacerbations, quantifying and stratifying the severity of the features
	To describe treatment attempts, taking in to account their efficacy as a whole and the impacts on the different aspect of the disease
	To report the safety profile of single treatment approaches in VEXAS patients
	To identify the better treatment approach tapered on the patient's features and disease characteristics
	To create standardized treatment protocols
	To assess any influence of the environmental background and the ethnic origin on the VEXAS syndrome phenotype;
	To assess the socioeconomic influence of the disease
	To monitor the cardiovascular risk in such patients
	To monitor the causes of death in VEXAS syndrome
Ancillary objectives	To design other pioneering studies according to the unmet needs arising from patients' management over time

### Inclusion/Exclusion Criteria

Patients carrying a *UBA1* gene somatic mutation and showing an inflammatory phenotype may be included in the Registry.

The patient has to give the written and informed consent after a careful explanation of the Project: the objectives of the Registry, the lack of implications on clinical management and treatment, the opportunity to withdraw the consent at any time, and the laws to comply with to guarantee patients' privacy, anonymity and security of data. Patients have to be ensured about the lack of consequences deriving from her/his will to participate or not to the study.

For patients unable to provide their consent, this should be given by legally authorized representatives, who must observe the study requirements for the entire duration of the study or until the consent withdrawal. In any case, the patient's assent is essential for patients aged ≥12 years.

No exclusion criteria or conditions are previewed to the enrolment.

### Online Data Collection

The Research Electronic Data Capture (REDCap) instrument has been used for data gathering and storing. REDCap is an electronic data collector produced at Vanderbilt University Medical Center (VUMC). It is currently located at the Virginia Commonwealth University (Award Number UL1TR002649). The employment of the REDCap platform is free to all the members of the *REDCap consortium*, which may benefit from using the tool in return for technical support. At current, take part in the *REDCap consortium* from the 4 continents over 5,600 worldwide institutions from 144 Countries already (9). To access the Registry, Principal Investigators and Site Investigators have to enter their own username and password. The Registry is available at the webpage https://aidanetwork.org/en/register/vexas. Data are kept on the servers of the University of Siena, Siena, Italy. Privacy is ensured for each Centre's data, with Principal and Site Investigators unable to access data collected in other Centers.

The Registry's browser interface provided for the data entry is entirely supplied in English in order to reduce the language barriers and facilitate the international data collection.

The retrospective assessment requires the collection of clinical and laboratory data referring to the symptoms of the disease at the onset, at the diagnosis, and at the enrollment into the Registry; clinical and laboratory data would be inserted referring to the start of each treatment performed, the 3-, 6- and 12-month visits and at the last assessment. On the other hand, follow-up visits will be added at the visits performed after the inclusion in the AIDA Registry; follow-up assessments should be filled in at least every year and at any change in the treatment strategy, as for the introduction of new drugs and posology changes. Socioeconomic data include variables embedded to assess the impact of VEXAS syndrome on the national health care system (access to primary care physician, specialist visits, laboratory examinations, imaging tests, access to emergency care and hospitalization) and on the working world (absenteeism and presenteeism).

The Investigators will be responsible for the own study data introduced in the online Registry and for the precision of the information accrued; the Principal Investigator is required to check for the accuracy of the data. The online access through personal username and password guarantees the security of the patients' information.

### Ethics

In June 2019 the Ethics Committee of the Azienda Ospedaliero-Universitaria Senese, Siena, Italy (Ref. N. 14951) granted the first national regulatory approval for the AIDA Project. After that, Centers experienced with diagnosis, clinical management, and treatment of autoinflammatory diseases have joined the AIDA Network from Europe, Middle East, Africa and America.

Patients' information is kept in accordance with the EU General Data Protection Regulations (GDPR) on the processing of personal data and the protection of privacy (2016/679/EU) (10), or other counterparts.

Regarding the patients' voluntary informed consent, the AIDA registries meet the recommendations from the Declaration of Helsinki. For minor patients aged ≥12 years the assent is also required when the patient is not competent to provide the consent. In these cases, parents/legal guardians have to provide their authorization to allow the patient's participation in the Project.

Consent for processing data for statistical or research purposes may be withdrawn at any time by either patients or Principal Investigators. In these cases, no further information will be captured; moreover, the patient has the right to obtain the complete erasure of all personal data already gathered in the Registry if required and notified to the study Promoter (University of Siena).

No financial remuneration is planned for patients or physicians for the study participation; in addition, there is no evidence of any billing relationships with the national health systems or insurance companies.

### Statistical Analysis

Statistical analysis will be based on the specific type and nature of data undergoing computation and will be performed according to the specific objectives of the studies conducted on behalf of the AIDA Network. In any case, the analysis will embrace general principles of descriptive statistics, correlations between groups and comparisons between subgroups. Details about statistics will be provided in the future papers obtained from data collected in the VEXAS Registry.

Principal Investigators and Site Investigators are encouraged to put forward their study proposals during the AIDA meetings. The data collected in a center may be analyzed by satellite centers independently from the other centers on condition that the AIDA Network appears among acknowledgments. On the contrary, the totality of data collected in the Registry will be managed by statistics and physicians involved in the network and selected based on their field of expertise.

## Results

The development and activation of this AIDA Registry is a first fundamental result of the AIDA Network. In this regard, an international registry dedicated to VEXAS syndrome is essential to extensively gather real-life data in a quick manner.

**Figure 1:**
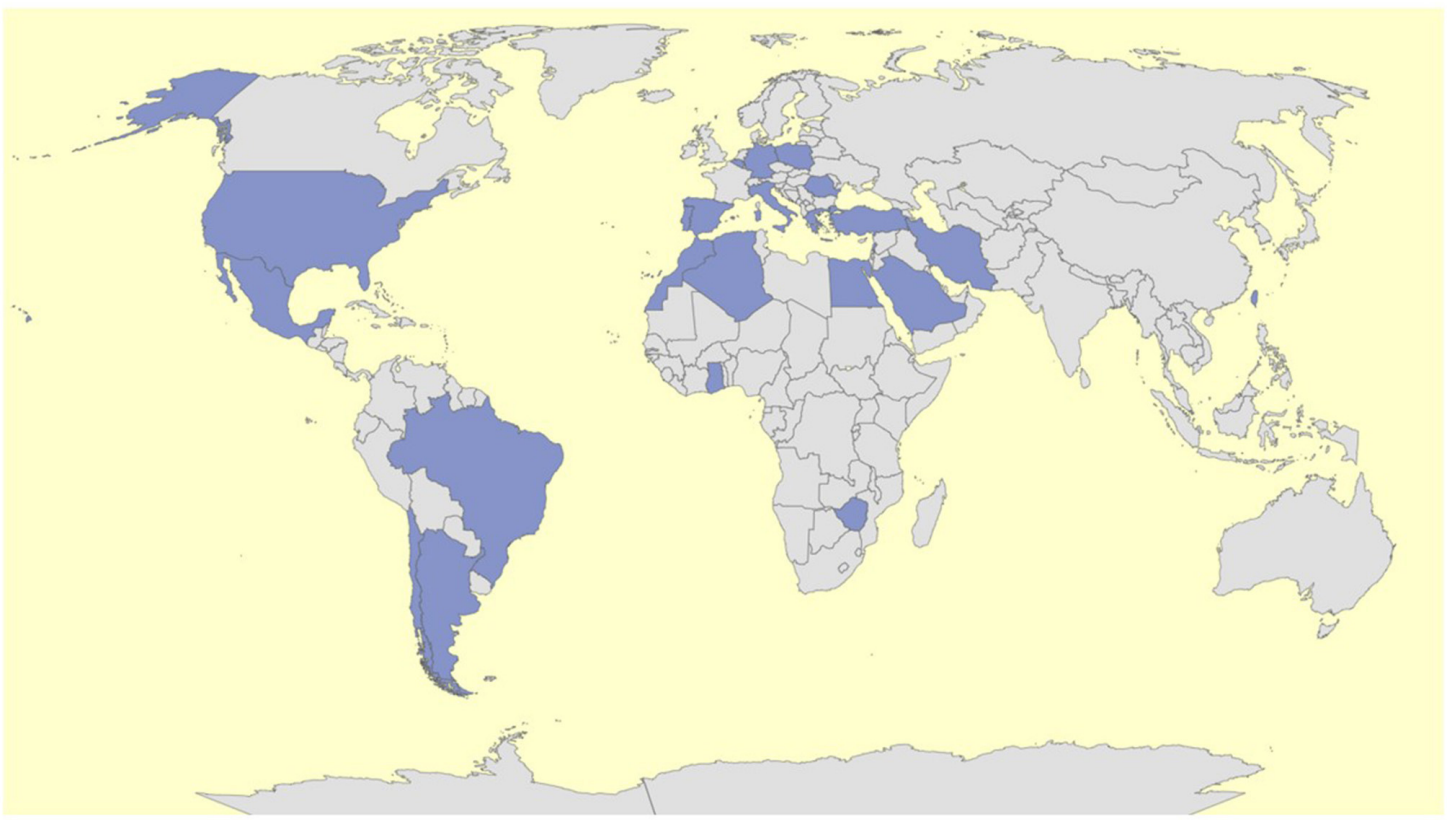
Worldwide distribution of the AIDA network on 22^nd^ of April 2022.

To date, 23 nations in 4 continents (Algeria, Argentina, Belgium, Brazil, Chile, Egypt, Germany, Ghana, Greece, Iran, Italy, Lebanon, Morocco, Mexico, Poland, Portugal, Romania, Saudi Arabia, Spain, Taiwan, Turkey, United States, Zimbabwe) have already joined the AIDA Network. The Figure 1 highlights the current (April 22^nd^, 2022) worldwide distribution of the AIDA Network.

Overall, 113 Centers around the world have joined the AIDA project for a total of 324 users (114 principal investigators, 205 site investigators, 2 lead investigators, 3 data managers). This Project was registered at ClinicalTrials.gov (ID: NCT05200715).

### Registry Development

When choosing the clinical variables constituting the Registry, information useful for a valuable knowledge of VEXAS syndrome was included, in order to quickly obtain data and comprehensively understand this new clinical entity in a relatively short time. For this reason, the registry was developed to comprehensively trace the entire clinical and therapeutic history of the patients enrolled. To date (April 22^nd^, 2022), the Registry contain 4,952 common data elements (each corresponding to a study variable) organized into 18 instruments. Thirteen of these instruments are specifically built to collect retrospective information, one instrument is dedicated to the prospective phase and 4 instruments should be used both to collect retrospective information and to describe any change starting from the time of the enrolment. Table 2 provides more detailed information about the instruments included in this Registry, the phases they refer to and the number of fields they include.

**Table 2:** Panel of instruments constituting the registry dedicated to subject with VEXAS (vacuoles, E1 enzyme, X-linked, autoinflammatory, somatic) syndrome; the number of common data elements are also provided along with the phase (i.e., retrospective/prospective) at which they should refer.

**Instruments**	**Fields**	**Retrospective/prospective phase**	**No. of mandatory fields**
Demographics	10	Retrospective phase	4
Consents	4	Retrospective phase	2
General information about VEXAS onset	10	Retrospective phase	2
VEXAS features up to the enrollment	148	Retrospective phase	3
Concomitant hematological disorders	23	Retrospective/prospective phase	0
Concomitant and associated diseases	19	Retrospective/prospective phase	1
Genetic information	6	Retrospective phase	1
Otherthan*UBA1* gene mutations	8	Retrospective phase	0
Laboratory data	156	Retrospective phase	5
Bone marrow evaluation	6	Retrospective phase	0
Cardiovascular risk	24	Retrospective/prospective phase	2
Past and current treatments	2	Retrospective phase	0
Corticosteroid monotherapy/main therapy–the retrospective phase	256	Retrospective phase	1
Treatment with cDMARD not associated to biotechnological agents–the retrospective phase	647	Retrospective phase	6
Treatment with small molecules not associated to biotechnological agents–the retrospective phase	1,271	Retrospective phase	12
Treatment with biotechnological agents–the retrospective phase	1,245	Retrospective phase	14
Follow-up visits–the prospective phase	1,093	Prospective phase	60
Death of the patient (to open only in case of patient's death)	4	Retrospective/prospective phase	0

Common data elements consist of demographic, clinical, instrumental, histological, laboratory, therapeutic and any other medical variable required to fully describe disease course. Many of these are shared with other AIDA registries dedicated to different autoinflammatory and non-infectious ocular diseases, to facilitate the merging of data among different Registries.

Each variable will require to be answered only in case it is useful according with the patient's clinical picture. This is allowed by a branching mechanism that will drive the opening of the answers only in case it will be necessary to complete a previously provided information. Therefore, only a small number of the 4,952 variables will appear to the investigators.

### Patients' Involvement

As for other autoinflammatory diseases, patients and patients' associations play a pivotal role in supporting data collection and the diffusion of the Project. Indeed, patients may stimulate Centers to join the Project, provide their own time for data collection, and supply patient's reported outcomes.

The associations of patients were invited to furnish patients' opinion about how to develop this AIDA Registry since the very beginning of the project. In particular, patients' point of view and impressions were required before creating variables and instruments of the Registry, while concerns, related to the use of sensitive data were solved together. In order to meet patients' expectations, a Registry focused on the different aspects of VEXAS syndrome was developed. In this way, clinical, laboratory, and therapeutic unmet needs will be widely investigated without giving preference to a single field.

The AIPF (*Associazione Italiana Febbri Periodiche*), the ANMAR (*Associazione Nazionale Malati Reumatici*) and the APMARR (*Associazione Nazionale Persone con Malattie Reumatologiche e Rare*) are currently giving their active support. To date, on Italian patients' organizations are included in the project; however, other international patient advocatory groups are about to join AIDA worldwide.

## Discussion

VEXAS syndrome is a very recently identified autoinflammatory disorder caused by acquired somatic mutations of *UBA1* gene in blood cells precursors. Despite the genetic origin, the epidemiological burden could be much higher than that characterizing hereditary periodic fevers. In addition to the knowledge gap about the prevalence, all the clinical aspects of the disease should be widely explored, especially in relation to the optimal therapeutic approach to use. As for other autoinflammatory diseases (11), treatment should be based on the specific disease manifestations, the long-term outcome, and complications arising over time. The use of a patient registry can dramatically facilitate these goals, allowing a better knowledge of the disease in a relatively shorter time.

Regardless of the real impact that this disease has in the population, VEXAS is to be considered a rare disease at present. Therefore, as for other autoinflammatory diseases (6–8), we have developed and launched a Registry capable of gathering the few real-life data available worldwide. While waiting for randomized controlled clinical trials, which are likely to take many years for their conduction, a registry dedicated to VEXAS syndrome can lead to the rapid collection of real-life data from a sufficiently large number of patients. This will allow the scientific community to achieve solid results that may be applied on VEXAS patients in daily clinical practice.

Of note, the AIDA Network is intended both to enable a broad population-based data collection and to enhance international collaboration, focusing the research efforts on international projects. In this regard, the first steps will be to reach a full knowledge about VEXAS clinical manifestations and their frequency, pointing out rare and atypical disease expressions. Furthermore, it is essential to describe the *UBA1* mutations really capable of determining VEXAS syndrome, highlighting pathogenic or likely pathogenic variants from benign polymorphisms. Actually, as for other genetic autoinflammatory conditions, low-penetrance variants and genotype-phenotype correlations may be described (12, 13).

It would be useful to search for any pathognomonic element capable to easily direct the diagnosis and genetic examination. In this regard, the presence of vacuoles and their number could be a central diagnostic factor, but their sensitivity and specificity should be confirmed on a wide number of patients (14). Similarly, bone marrow biopsy and aspirate can provide diagnostic or prognostic data that should be clarified.

Another focus of research should be to comprehensively disclose all the possible expressions of long-term hematologic involvement, revealing any predictive factors and complications. To date, it is well-known that VEXAS patients often present a hematological involvement with myelodysplastic syndrome, monoclonal gammopathy with unknown significance (MGUS), macrocytic anemia, and thrombocytopenia. However, myeloid malignancies are also frequently described in such patients (2, 15). These data must be confirmed and expanded in large cohorts, while a proper long-term follow-up should reveal all the various hematological aspects.

If VEXAS syndrome is only little known as a whole, the lesser-known aspect is the proper therapeutic approach. Many treatments have been tested in VEXAS syndrome, including glucocorticoids, conventional disease-modifying antirheumatic drugs (cDMARDs), azacytidine, biologically targeted agents and janus kinase (JAK) inhibitors. Except for corticosteroids, which are especially useful at high dosage, preliminary data show a significant interindividual variability in the effectiveness of these therapeutic strategies (1, 16). Therefore, identifying the better treatment approach based on the patient's features could allow the optimal treatment in the perspective of a personalized medicine. Similarly, the identification of the best dosages and the assessment of long-term safety profile represent indispensable goals to ensure a correct management.

As for other AIDA registries (6–8), the assessment of the socioeconomic influence of the disease on the national healthcare systems, on patients' social role and job impact represent an intriguing subject of analysis. Other objectives will be identified based on the challenges that clinical practice and scientific research will bring forward in the coming years. The Registry benefits from a remarkable plasticity and it may adapt to changes that will be required according to future acquisitions. In addition, the registry boasts the capability to communicate with other present or future registries dedicated to VEXAS syndrome; this will further enhance research projects through the merging of collected information.

Noteworthy, a new online tool defined as “AIDA for patients” is under development. “AIDA for patients” is an instrument primarily aimed at including patients in the network in terms of diffusion of the project, sharing of research strategies, outreach to physicians in the various centers toward a better and wider enrollment, and involvement of the patients themselves in providing their own data.

Since this disorder has been discovered only in recent times, there are not yet associations specifically dedicated to VEXAS syndrome; for this reason, existing associations of patients with rare rheumatological diseases have been involved.

The AIDA Registry for patients with VEXAS syndrome shows the typical limitations of observational studies. In particular, the completeness and accuracy of information accrued in the Registry accounts for the main issues of the retrospective phase. Furthermore, there is no obligation to consecutively enroll all the patients followed in the AIDA Centers, and this may cause an unintended selection bias. Enrolling patients in the Registry needs much time and attention, especially when the patient's history is remarkably long due to the complex clinical framework and numerous treatments approaches. Therefore, both investigators and patients enrolled have to be sensitized as to provide their time for the study purposes. This is especially true for the retrospective phase, which requires from 1–3 h for a complete data collection. Conversely, the prospective phase does not affect substantially physicians and patients' time, as 10 min are required to fill-in the follow-up page. Beyond these limitations, this Registry represents an unvaluable tool to fully understand the disease in terms of clinical management and treatment. Furthermore, the Registry may be a source for patients' enrolment in future randomized clinical trials.

## Conclusion

The AIDA international Registry dedicated to patients affected by VEXAS syndrome has been made available for data collection. Joining the AIDA project in reference to the VEXAS Registry will allow the achievement of a comprehensive knowledge about this new disease in a relatively short time. The final goal of this Project will be to conduct observational and prospective studies leading to real-world evidence to be applied in the everyday clinical practice for patients with VEXAS syndrome.

## Ethics Statement

The studies involving human participants were reviewed and approved by Ethics Committee of the Azienda Ospedaliero-Universitaria Senese, Siena, Italy (Ref. N. 14951). Written informed consent to participate in this study was provided by the participants' legal guardian/next of kin.

## Author Contributions

AV wrote the first draft of the manuscript, conceived and designed the study, and the registry for VEXAS syndrome. VC, FD, JH-R, MF, SM, AT, ST, and EC actively enrolled patients in the registry for VEXAS syndrome. GR, GL, IA, RP, DY, MC, AMar, TG, FLT, PR, EA, EW-S, ED, PSfi, MGo, GEm, MM, RG, FCi, GC, DA-I, CL, VS, MP, AS, DO-B, RI, EB, FF, PP, AP, GEs, AMai, GS, AI, FS, PSfr, FM, MAle, JM, GH, NA, FLG, AG, AO, SA-M, SE, and SG were the principal investigators of centers actively enrolling in the AIDA registries. IV, MT, AMah, MAlz, AL, FR, FCard, KJ-R, FCr, MD, MGh, CG, JS, ID, CF, HG, AF, AC, MB, and FCaro were included in the authorship as investigators from to the three top contributor centers for any of the other AIDA registries. GT, AR, BF, and MB actively contributed to the launching of the registry dedicated to VEXAS syndrome. AB involved in the technical management of the platform and registries. DR took care of the final revision of the manuscript. LC conceived and designed the study and accounted for AIDA registries coordinator. All authors contributed to the article and approved the submitted version.

## Conflict of Interest

The authors declare that the research was conducted in the absence of any commercial or financial relationships that could be construed as a potential conflict of interest.

## Publisher's Note

All claims expressed in this article are solely those of the authors and do not necessarily represent those of their affiliated organizations, or those of the publisher, the editors and the reviewers. Any product that may be evaluated in this article, or claim that may be made by its manufacturer, is not guaranteed or endorsed by the publisher.
